# Diagnostic Efficacy of FAPI-PET/CT Versus [^18^F]FDG-PET/CT in Upper-Abdominal Malignancies: A Systematic Review and Meta-Analysis

**DOI:** 10.3390/diagnostics16040520

**Published:** 2026-02-09

**Authors:** Hao Huang, Betül Altunay, Laura Schäfer, Christian Boy, Dirk von Mallek, Felix M. Mottaghy, Susanne Lütje

**Affiliations:** 1 Department of Nuclear Medicine, University Hospital RWTH Aachen, 52074 Aachen, Germany; hhuang@ukaachen.de (H.H.); baltunay@ukaachen.de (B.A.); laschaefer@ukaachen.de (L.S.); cboy@ukaachen.de (C.B.); dvonmallek@ukaachen.de (D.v.M.); fmottaghy@ukaachen.de (F.M.M.); 2 Center for Integrated Oncology (CIO), University Hospital RWTH Aachen, 52074 Aachen, Germany; 3 Department of Radiology and Nuclear Medicine, Maastricht University Medical Center, 6229 HX Maastricht, The Netherlands

**Keywords:** fibroblast activation protein, PET/CT imaging, [^18^F]FDG, pancreatic cancer, liver cancer, gastric cancer

## Abstract

**Background**: Radiolabeled fibroblast activation protein inhibitors (FAPIs) have emerged as novel radiopharmaceutical agents for tumor diagnosis. Compared with [^18^F]fluoro-2-deoxy-D-glucose ([^18^F]FDG), which reflects glucose uptake in metabolically active regions, FAPIs mainly bind to the fibroblast activation protein (FAP), which is highly expressed in tumor-associated fibroblasts, forming a pronounced signal. Several studies suggested potential superiority of FAPI tracers above [^18^F]FDG-based imaging in a variety of tumor entities. In this systematic review, we focus on the comparison of FAPI-PET/CT and [^18^F]FDG-PET/CT in upper-abdominal tumors. **Methods**: Original research published from 1 January 2021 to 22 December 2024 was collected from the PubMed and Web of Science databases (CRD42025648267). This research included only clinical studies, excluding conference abstracts and case reports. The risk of bias was assessed with the QUADAS-2 tool, and all evaluation steps performed independently by three independent reviewers. A systematic quality assessment of the included studies was conducted based on the imaging performance of FAPI-PET/CT and [^18^F]FDG-PET/CT for pancreatic, liver, and gastric cancers. The meta-analysis used relative risk (RR) as the effect size, with bias assessed via the Peters test (*p*-value > 0.05). Cochran’s Q test and I-squared value are used to comprehensively evaluate the magnitude of heterogeneity. Analyses and data visualization were performed in R language. **Results**: The database search identified 3272 articles. After screening, 31 studies were included in this analysis. The original studies enrolled 1377 participants (M/F: 850/527; ages predominantly between 50 and 70). Of these, 939 patients were ultimately diagnosed with tumors (five cancer subtypes) and included in this analysis. Meta-analysis results showed that FAPI-PET/CT significantly surpassed [^18^F]FDG-PET/CT in the detection of primary lesions (RRs = 1.20 and 1.17), lymph nodes (RRs = 1.18 and 1.24), distant metastases (RRs = 1.22 and 1.51), peritoneal metastases (RRs = 1.31 and 2.22), and bone metastases (RRs = 1.16 and 1.23). The two imaging methods exhibit clear differences in diagnostic performance (sensitivity: 98% vs. 79%; specificity: 83% vs. 87%), and FAPI-PET/CT demonstrates high and stable diagnostic performance (RRs = 1.20 and 1.17). **Conclusions**: Compared with [^18^F]FDG-PET/CT, FAPI-PET/CT demonstrates significant advantages in detecting primary lesions, lymph nodes, distant metastases, and peritoneal and bone metastases in pancreatic, liver, and gastric cancers (RR > 1.0). Overall, FAPI-PET/CT shows better diagnostic performance (RR > 1.0).

## 1. Introduction

Malignant tumors of the upper abdomen, particularly pancreatic, hepatic, and gastric cancers, remain a major cause of cancer-associated morbidity and mortality worldwide [1]. Accurate diagnostic imaging is essential for detecting these malignancies at earlier stages, guiding therapeutic interventions, and assessing treatment responses [2]. Among the available imaging modalities, PET/CT with [^18^F]FDG has emerged as an integral component in routine oncologic evaluation, leveraging the principle of elevated glucose metabolism in tumor cells [3].

Despite its utility, [^18^F]FDG-PET/CT faces well-known limitations in certain clinical scenarios. First, physiologically high [^18^F]FDG uptake in stomach, liver, and pancreatic tissues can hinder visualization of neoplastic lesions and reduce overall sensitivity for tumor detection [4]. Second, tumors with low or heterogeneous glucose metabolism, such as certain neuroendocrine and well-differentiated neoplasms, may yield false-negative findings and thus limit the modality’s diagnostic accuracy [5,6]. Finally, inflammatory conditions and recent therapeutic interventions (e.g., surgery or radiotherapy) can increase local [^18^F]FDG uptake, complicating the differentiation between malignant and benign processes [7,8].

To solve these diagnostic challenges, novel radiotracers targeting alternative tumor-associated pathways have gained attention. Since 2018, FAPI-based tracers, such as ^68^Ga-FAPI-46 and ^18^F-FAPI-2, have been introduced [9,10]. FAPI-PET/CT has demonstrated promising potential in identifying various tumors, including those of the upper abdomen, by binding to cancer-associated fibroblasts independently of local glucose metabolism [11]. Notably, up to 90% of pancreatic, hepatic, and gastric tumors overexpress FAP, whereas approximately 20–35% of these malignancies exhibit relatively low [^18^F]FDG avidity [12,13,14,15]. In addition, FAPIs demonstrate certain advantages in the clinical diagnosis of rare tumors [16].

Given the relatively high physiological [^18^F]FDG uptake in upper-abdominal structures and the limited potential of [^18^F]FDG-PET/CT in distinguishing benign from malignant lesions in this region, a systematic evaluation of the diagnostic efficacy of FAPI-PET/CT is warranted [17,18,19,20,21,22,23,24,25,26,27,28,29,30,31,32,33,34,35,36,37,38,39,40,41,42,43,44,45,46,47]. Compared with previous similar studies, this review included a larger number of upper-abdominal tumor samples and compared the diagnostic performance of FAPI-PET/CT and [^18^F]FDG-PET/CT. Within this metabolically challenging topography, our meta-analysis focuses on comparing FAPI-PET/CT and [^18^F]FDG-PET/CT in gastric, hepatic, and pancreatic cancers for the diagnostic accuracy of primary tumors and metastatic foci, and potential advantages for clinical decision-making in the management of malignancies.

## 2. Materials and Methods

### 2.1. Search Strategy

This study was conducted following the PRISMA guidelines, and each item in the PRISMA checklist and flow diagram was systematically reviewed for alignment with the reported content (https://www.prisma-statement.org/prisma-2020-checklist). This study has been registered within the PROSPERO framework (https://www.crd.york.ac.uk/prospero/), with the registration number: CRD42025648267. Researchers conducted a systematic search using the following keyword combinations: (‘FAPI’ or ‘fibroblast activation protein’) and (‘upper abdominal’); (‘FAPI’ or ‘fibroblast activation protein’) and (‘liver’ or ‘HCC’ or ‘ICC’); (‘FAPI’ or ‘fibroblast activation protein’) and (‘pancreatic’); and (‘FAPI’ or ‘fibroblast activation protein’) and (‘gastric’ or ‘stomach’). The search was performed for clinical research articles published in PubMed (https://pubmed.ncbi.nlm.nih.gov/) and Web of Science (https://www.webofscience.com/wos) from 1 January 2021 to 22 December 2024 (accessed on 22 December 2024).

### 2.2. Inclusion Criteria

Two professional medical specialists systematically screened the titles and abstracts against the following criteria, with a third medical specialist assisting in the screening process as necessary. Studies eligible for inclusion involved pancreatic tumors, gastric tumors, or liver tumors diagnosed using FAPI-PET/CT imaging, and provided corresponding [^18^F]FDG-PET/CT reference data. The outcome events for the selected studies included at least histopathological results and confirmation via surgical exploration, and a minimum imaging follow-up period of three months. Included studies provided relevant data for the analysis of primary outcomes. Additionally, study types such as case reports, conference abstracts, reviews, or commentaries were excluded. Preclinical studies not involving human subjects and clinical studies with a sample size of less than 10 patients were also excluded.

### 2.3. Data Collection

This study extracts basic information (including but not limited to authors and years), patient data (including but not limited to age, gender, and types of tumors), and imaging data (lesion detection rates, SUV_max_, and data related to diagnostic performance, etc.) from the literature that meets the inclusion criteria. Three reviewers independently performed data collection with manual reviews. In several studies, the missing data were not included in this analysis.

### 2.4. Analysis Methods

Due to the included study objectives differing in age, gender, and disease stage, we employed a combination of random-effects and fixed-effects approaches for the analysis. Random-effects models account for potential heterogeneity in studies and, thus, reflect the distribution of true effect sizes. A random-effects model was applied if the heterogeneity test indicated substantial inconsistency (I^2^ > 50% or Cochran’s Q test with *p*-value < 0.10). If I^2^ < 50% and Cochran’s Q test *p*-value was > 0.10, a fixed-effects model was used. Furthermore, to ensure the accuracy of the analysis, researchers conducted subgroup analyses and sensitivity analyses to compare the performance of FAPI-PET/CT with [^18^F]FDG-PET/CT in tumor diagnosis. In the sensitivity analysis of the effect size, a leave-one-out approach was used to assess whether the overall effect changed, and the results were presented as a forest plot. Additionally, cumulative effects were observed to evaluate the performance of FAPI-PET/CT in tumor diagnosis. R is an open-source software that is widely used in medical statistics. In this study, all data analyses and visualizations were performed using R (version 4.4.2), with the ‘meta’ and ‘meta4diag’ packages. The final data visualization is shown as a forest plot to summarize the effect estimates in the studies.

### 2.5. Risk of Bias

This study was conducted in accordance with the PRISMA 2024 framework. Two reviewers conducted an independent assessment of the bias risk in the literature according to the QUADAS-2 tool. Whenever disagreements arose, a third reviewer was brought in to assess the bias risk. Each of the included studies were summarized according to typical ‘risk of bias’ domains, including patient selection, index test, reference standard, flow, and timing. Additionally, the Peters test was used to assess publication bias in the included studies, and the results were visualized with a funnel plot. Where relevant, additional considerations, such as small human cohort size, were noted.

## 3. Results

### 3.1. The Literature Selection

**Figure 1:**
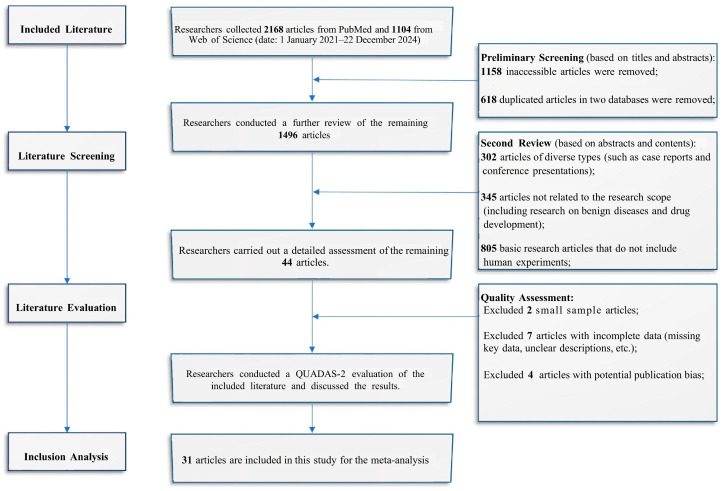
Flowchart of the selection process for the literature included in this study.

Researchers retrieved 3272 articles published from two databases, and 44 articles relevant to this study were identified (Figure 1). Based on the exclusion criteria, a total of 31 articles were included in this study (Supplementary Table S1). Additionally, the included studies provided precise data on tumor lesion counts. Excluded were studies with incomplete data and small sample sizes, as well as studies with potential publication bias.

### 3.2. Publication Bias Assessment and Systematic Review

Based on the QUADAS-2 evaluation criteria, researchers evaluated the included studies (Supplementary Figure S1). Among the 31 included studies, none were rated high risk on any of the four evaluation criteria, 18 were low risk, and the remaining 13 were moderate risk. The study evaluated publication bias in patient and lesion data, with the funnel plot showing that the *p*-value is greater than 0.05, indicating the absence of publication bias in the included data (Supplementary Figure S2). The L’Abbé graph macroscopically demonstrates that the diagnostic performance of FAPIs is superior to that of [^18^F]FDG.

Subsequently, researchers conducted statistics of the sample data from the included studies (Table 1). All studies have progressed to the clinical practice stage. The original study enrolled 1377 participants (M/F: 850/527; ages predominantly between 50 and 70). The proportion of male patients was significantly higher in the original study. In this study, a total of 939 patients were included, with 943 primary lesions and 1786 distant metastatic lesions compared. In total, five cancer subtypes were included in the analysis: pancreatic cancer (PC), consisting of pancreatic ductal adenocarcinoma only; liver cancer, comprising hepatocellular carcinoma (HCC) and intrahepatic cholangiocarcinoma (ICC); and gastric cancer (GC), including gastric adenocarcinoma and gastric stromal tumors. Most of the studies originate from China. Additionally, the present study included 18 prospective studies and 13 retrospective studies.

### 3.3. Meta-Analysis

#### 3.3.1. Comparative Analysis of FAPI-PET/CT and [^18^F]FDG-PET/CT Data Based on Patients

**Figure 2:**
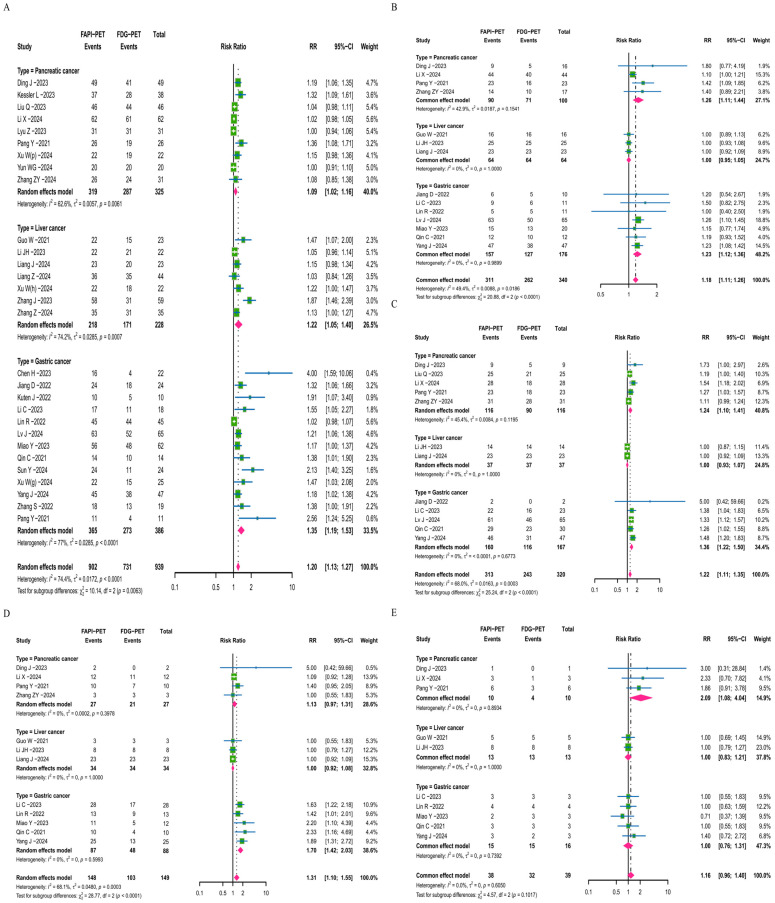
Forest plots based on patient data for FAPI-PET/CT and [^18^F]FDG-PET/CT illustrate tumor detection capabilities, including general tumor detection (**A**), lymph node metastases (**B**), distant metastases (**C**), peritoneal metastases (**D**), and bone metastases (**E**) for FAPI-PET/CT and [^18^F]FDG-PET/CT [17,18,19,20,21,22,23,24,25,26,27,28,29,30,32,33,35,37,38,39,41,42,43,44,45,46,47].

The results from the research based on patient diagnostic data demonstrate that FAPI-PET/CT outperforms [^18^F]FDG-PET/CT in the area of tumor detection, as well as in identifying distant metastasis and lymph node metastasis, with significant differences between the two imaging methods (Supplementary Table S2). Furthermore, the sensitivity analysis showed that the data for each group were stable (Supplementary Figure S3). Based on the data analysis results of patients diagnosed with tumors, the overall RR value is found to be 1.20, with a 95% confidence interval (CI) of 1.13–1.27 (Figure 2A). This indicates that FAPI-PET/CT shows significant advantages in upper-abdominal tumor diagnosis. Moreover, the results of the subgroup analysis indicate that the RR values are greater than 1.0 across all cancer subgroups, further supporting the advantage of FAPI-PET/CT in tumor diagnosis among different upper-abdominal types of cancers. We further analyzed and compared the data of patients with lymph node metastasis and distant metastasis. The overall RR values for FAPI-PET/CT compared to [^18^F]FDG-PET/CT are found to be 1.18 and 1.22, with CIs of 1.11–1.26 and 1.11–1.35, respectively (Figure 2B,C). In the subgroup analysis of distant metastasis and lymph node metastasis, the data to support the advantage of FAPI-PET/CT are not significant in the liver cancer subgroup (RR = 1.0); however, for pancreatic cancer and gastric cancer, the data show significant advantages of FAPI-PET/CT over [^18^F]FDG-PET/CT, with RR values for lymph node metastasis at 1.26 and 1.23, and for distant metastasis at 1.24 and 1.36, respectively. Considering the advantages of FAPI-PET/CT in peritoneal and bone imaging, the research team conducted a separate analysis and comparison for patients with peritoneal and bone metastases (Figure 2D,E). The result shows that FAPI-PET/CT has a diagnostic advantage over [^18^F]FDG-PET/CT in patients (RRs = 1.31 and 1.16).

#### 3.3.2. Comparative Analysis of FAPI-PET/CT and [^18^F]FDG-PET/CT Data Based on Lesions

**Figure 3:**
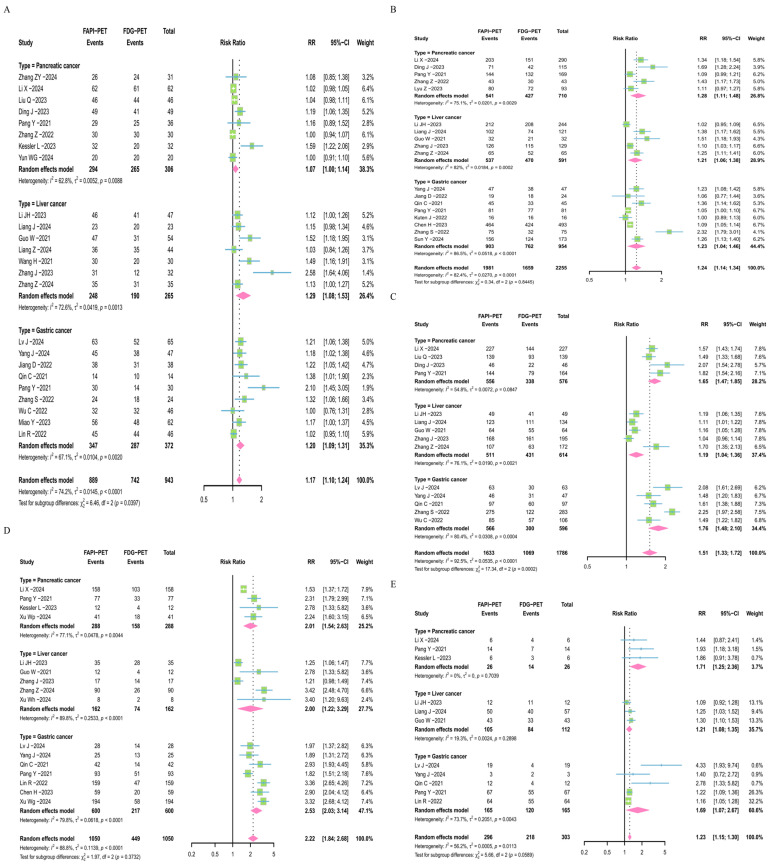
Forest plots based on lesion data for FAPI-PET/CT and [^18^F]FDG-PET/CT. Forest plot of the number of primary tumor lesions (**A**), lymph node metastatic lesions (**B**), distant metastatic lesions (**C**), peritoneal metastatic lesions (**D**), and bone metastatic lesions (**E**) detected by FAPI-PET/CT and [^18^F]FDG-PET/CT [17,18,19,20,21,22,23,24,26,27,28,29,30,33,34,35,36,39,43,44,46].

The researchers collected lesion data and conducted a sensitivity analysis on the subgroups, showing that the RR values remain stable (Supplementary Table S3 and Figure S4). In the analysis of primary tumor lesions among cancer subgroups, the RR values are all greater than 1.0 (CI: 1.10–1.24) (Figure 3A). Moreover, FAPI-PET/CT demonstrates an even more pronounced advantage over [^18^F]FDG in diagnosing metastatic lymph node lesions (RR = 1.24, CI: 1.14–1.34) (Figure 3B). In the analysis of distant metastatic lesions, consistent results were obtained, showing a significant difference between FAPI-PET/CT and [^18^F]FDG-PET/CT (RR = 1.51, CI: 1.33–1.72), indicating that FAPI-PET/CT detects more distant metastatic lesions (Figure 3C). Additionally, the meta-analysis results for 1050 peritoneal metastatic lesions and 303 bone metastatic lesions indicate that FAPI-PET/CT also shows a clear advantage in metastatic lesions (RRs = 2.22 and 1.23, CIs: 1.84–2.68 and 1.15–1.30) (Figure 3D,E).

#### 3.3.3. Comparison of SUV_max_ Data Between FAPI-PET/CT and [^18^F]FDG-PET/CT Lesions

**Figure 4:**
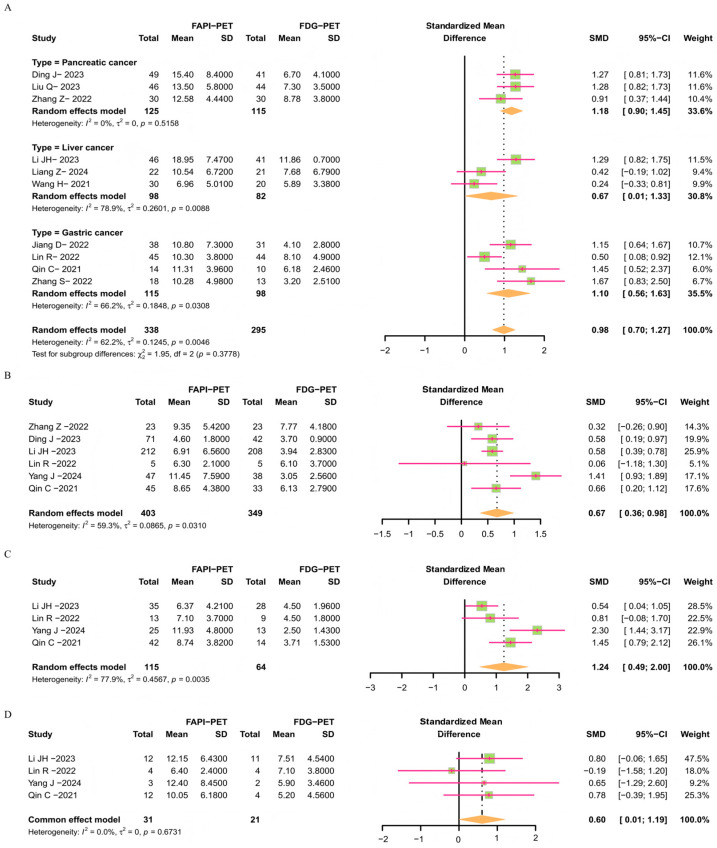
Forest plots based on lesion SUV_max_ data for FAPI-PET/CT and [^18^F]FDG-PET/CT. Forest plot of the SUV_max_ data for tumor lesions (**A**), lymph node metastatic lesions (**B**), peritoneal metastatic lesions (**C**), and bone metastatic lesions (**D**) detected by FAPI-PET/CT and [^18^F]FDG-PET/CT [19,20,22,25,28,29,31,33,34,35].

Researchers collected SUV_max_ values of tumors from the included literature (Supplementary Table S4 and Figure S5). The meta-analysis results indicate that there are significant differences between FAPI-PET/CT and [^18^F]FDG-PET/CT in SUV_max_ for tumor lesions and lymph node metastatic lesions, with standardized mean difference (SMD) values all greater than 0.5 (Figure 4A,B). Particularly for the imaging data of upper-abdominal tumor lesions, the results show an SMD value of 0.98 (CI: 0.70–1.27), indicating a significant advantage of FAPI-PET/CT imaging over [^18^F]FDG-PET/CT. Additionally, the comparison of SUV_max_ values for peritoneal and bone metastatic lesions also acquired consistent results (RRs = 1.24 and 0.60) (Figure 4C,D).

#### 3.3.4. Analysis of Diagnosing Performance of FAPI-PET/CT and [^18^F]FDG-PET/CT

**Figure 5:**
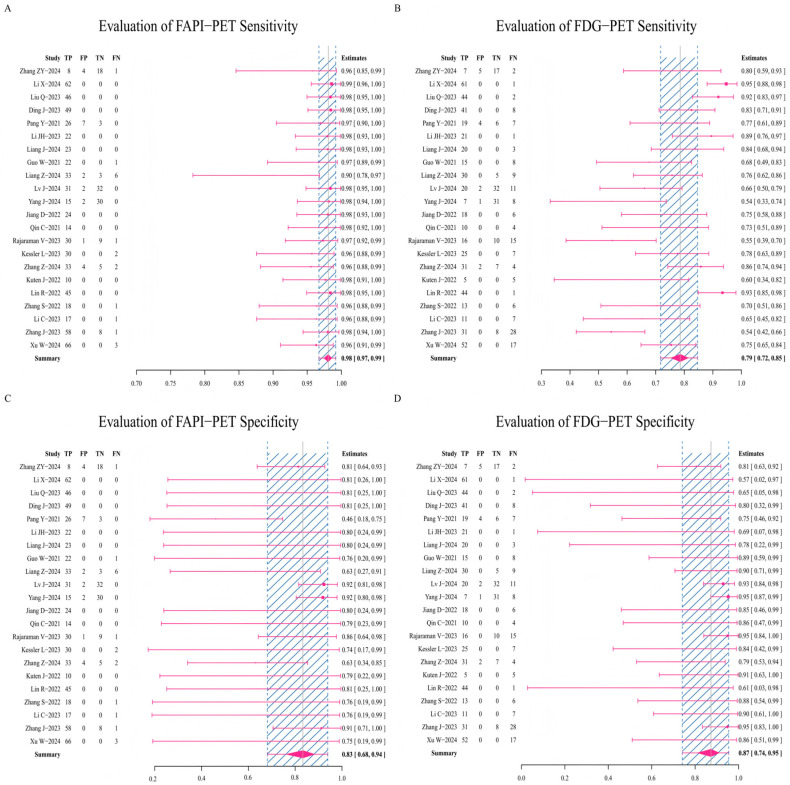
Forest plots of diagnostic performance based on the sensitivity and specificity of FAPI-PET/CT and [^18^F]FDG-PET/CT (TP: true positive; FP: false positive; TN: true negative; FN: false negative). Forest plot of the sensitivity performance for FAPI-PET/CT (**A**) and [^18^F]FDG-PET/CT (**B**); forest plot of the specificity performance for FAPI-PET/CT (**C**) and [^18^F]FDG-PET/CT (**D**) [17,18,19,20,22,23,24,25,26,27,28,29,30,32,33,35,38,40,42,43,44,45].

Researchers summarized the diagnostic performance data of FAPI-PET/CT and [^18^F]FDG-PET/CT. In the sensitivity data comparison analysis, the result for FAPI-PET/CT shows a stable sensitivity of around 98% (Figure 5A). In contrast, the sensitivity of the [^18^F]FDG-PET/CT imaging method just reaches 79% (Figure 5B). When further comparing the specificity of the two imaging methods, [^18^F]FDG-PET/CT shows a slight advantage over FAPI-PET/CT, with FAPI-PET/CT maintaining a specificity of approximately 83% (Figure 5C), while [^18^F]FDG-PET/CT achieves 87% specificity (Figure 5D). Overall, the results suggest that in terms of diagnostic performance data, FAPI-PET/CT has an advantage over [^18^F]FDG-PET/CT in terms of overall accuracy.

#### 3.3.5. FAPI-PET/CT Cumulative Meta-Analysis

**Figure 6:**
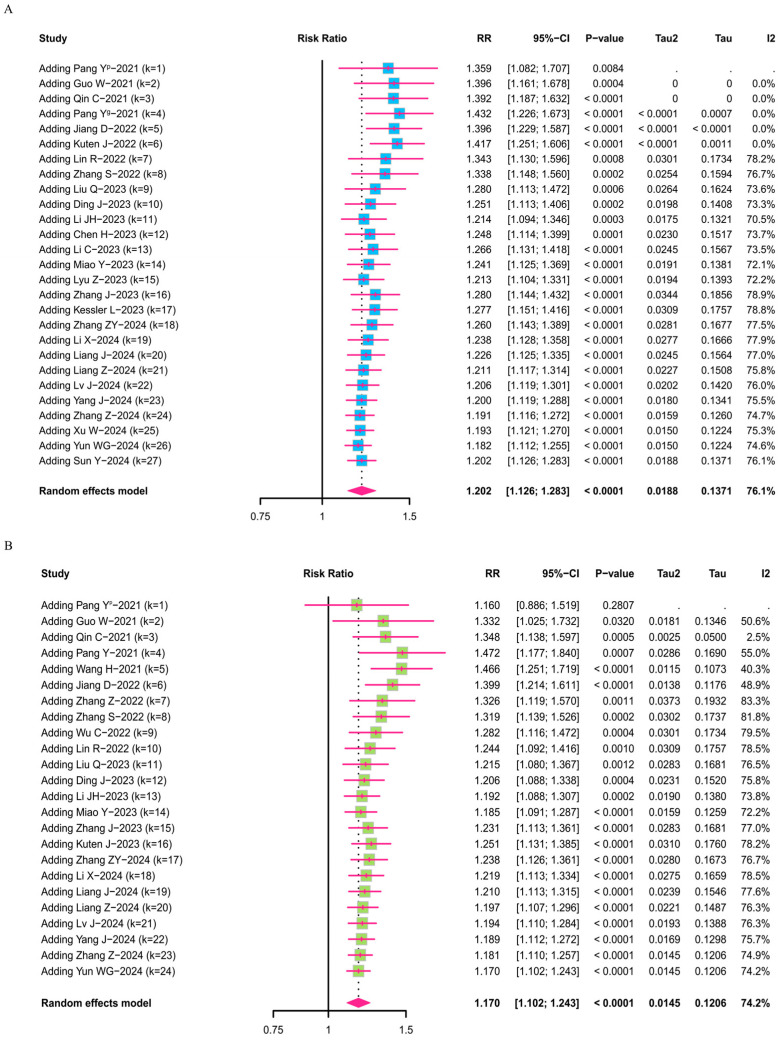
Cumulative meta-analysis forest plots sorted by the publication date of the included literature. Cumulative meta-analysis forest plot based on the number of tumor patients (**A**) and tumor lesions (**B**) diagnosed using FAPI-PET/CT and [^18^F]FDG-PET/CT [17,18,19,20,21,22,23,24,25,26,27,28,29,30,32,33,35,37,38,39,41,42,43,44,45,46,47].

The researchers conducted a cumulative meta-analysis of the diagnostic data for FAPI-PET/CT based on the number of patients, following the chronological order of the included literature. The results of the analysis indicate that, over time, the CI for FAPI-PET/CT compared to [^18^F]FDG-PET/CT stabilized between 1.12 and 1.29, while the RR value stabilized at 1.20 (Figure 6A). Similarly, consistent results are obtained in the analysis based on tumor lesion data, showing that FAPI-PET/CT has a significant advantage over [^18^F]FDG-PET/CT in diagnosing upper-abdominal tumors (such as pancreatic, liver, and gastric cancers) with an RR value of 1.17 (CI: 1.10–1.25) (Figure 6B). In addition, all data and corresponding content-related items have been presented in the PRISMA Checklist (Supplementary Table S5).

## 4. Discussion

The present systematic review and meta-analysis provides an integrated view of the current evidence comparing FAPI-PET/CT with the established [^18^F]FDG-PET/CT in pancreatic, hepatic and gastric malignancies. These three entities were intentionally pooled because they share several imaging challenges, including the inherently high physiological [^18^F]FDG uptake in adjacent upper-abdominal organs and a frequent propensity for desmoplastic stromal reactions. In addition, all three cancers exhibit meaningful levels of FAP expression and display similar metastatic routes, particularly toward the peritoneum. Single-tracer studies are more sensitive to differences in individual study design. Therefore, head-to-head comparison studies within the same cohort can better control biological variability and technical differences. As previously mentioned, this study used the RR values of the diagnostic rate between two tracers as the indicators; data from single-tracer studies were not included.

Across 939 patients and more than 1700 distant metastatic lesions, FAPI imaging yielded higher detection rates for primary tumors, lymph-node involvement and, most strikingly, peritoneal and bone metastases. While the pooled relative-risk gain for primary lesions was modest (~10–30%), it became more pronounced for distant disease, most notably for peritoneal, where the lesion-based RR values approached 1.51 and 2.22, respectively. Simultaneously, the research data indicate that the advantages of FAPI-PET/CT in the subgroup of liver cancer are less pronounced compared to those observed in pancreatic and gastric cancers. This attenuation may reflect the lower and more heterogeneous FAP expression reported for hepatocellular and cholangiocellular tumors, whereas pancreatic and gastric adenocarcinomas typically contain a dense desmoplastic, FAP-rich stroma [48,49,50,51]. The analysis of diagnostic performance data indicates that FAPI-PET/CT significantly surpasses [^18^F]FDG-PET/CT in terms of sensitivity, while showing some shortcomings in specificity. High pooled sensitivity was accompanied by a specificity of about 83%, slightly below that of [^18^F]FDG-PET/CT (87%). In addition, FAPI-PET/CT scans showed higher SUV_max_ values and, therefore, better lesion-to-background contrast, yet a higher SUV does not automatically equate to greater accuracy, particularly when benign fibroblast-rich conditions such as post-surgical scarring or granulomatous disease are present [52,53].

FAPI-PET/CT imaging targets fibroblast activation, offering a tumor detection method independent of glucose metabolism [54]. This approach is particularly suitable for patients with diabetes, inflammation, infection, or recent surgery or radiation therapy [7,54]. However, non-specific uptake, high costs, and limited tracer availability may temper enthusiasm. In the diagnostic application of upper-abdominal tumors, it is necessary to correlate imaging findings with clinical signs and, where necessary, histological confirmation. Further exploration of stable and easily synthesized probes is worth considering to enhance cost-effectiveness. In addition, multicenter prospective studies focusing on FAPI-PET/CT in the upper-abdominal tumor domain should be established to facilitate a diagnostic–therapeutic integrated approach. Recent systematic reviews indicate that FAPI-RLT holds broad promise in oncology, particularly for patients with abdominal cancers [55]. Moving forward, future research should emphasize standardization and personalization to enable clinical implementation of FAPI-PET/CT.

Several limitations must be acknowledged. Despite efforts to reduce various biases (publication bias and selection bias) in this study, the fixed nature of meta-analysis inevitably subjects it to such influences [56]. Almost all included studies originated from single-center cohorts, and the heterogeneity in study design, tracer variants, and reference standards was substantial. The random-effects model was used to account for between-study heterogeneity and to provide a more robust overall effect estimate. However, because potential moderators were difficult to quantify with the available data, a meta-regression to explore intrinsic factors could not be performed. Consequently, the results are generalizable only to populations with age ranges, exposure standards, and follow-up periods similar to those in the included studies. This study focused on PET parameters alone, without other imaging modalities, and histopathological heterogeneity was not evaluated. The studies retrieved to date mainly focused on FAPI-04, with fewer reports on FAPI-42 and FAPI-74. Due to limited head-to-head comparison data for these tracers, separate analyses for FAPI-42 and FAPI-74 were not performed in this study. Moreover, most investigations performed FAPI-PET/CT up to 14 days after [^18^F]FDG-PET/CT; disease dynamics during this interval could have influenced comparative lesion counts. The overall sample size remains modest, and some lesion subgroups, particularly bone metastases, were small. Additionally, the included studies demonstrated a higher proportion of male cases, which can be explained by the epidemiological trend [57]. From a clinical standpoint, our findings support the use of FAPI-PET/CT in scenarios where [^18^F]FDG uptake is known to be variable or when accurate assessment of peritoneal dissemination is pivotal [58]. In liver tumors, the incremental benefit appears moderate and should be weighed against additional costs and scanner time. Compared with PET/CT, PET/MRI offers superior soft-tissue resolution. For certain pancreatic and intrahepatic lesions, PET/MRI may provide more precise delineations of the lesion margins and extent. This study included three small studies with PET/MRI imaging, and the sensitivity analyses of these studies showed no influence on the results. The comparative study of these two imaging modalities remains a topic for future work. Prospective, multicenter trials with standardized protocols and robust clinical endpoints are needed to validate these preliminary observations, and to clarify whether the higher lesion yield of FAPIs translates into improved patient outcomes and cost-effectiveness [59].

## 5. Conclusions

FAPI-PET/CT imaging demonstrates higher accuracy than [^18^F]FDG-PET/CT in detecting primary lesions, lymph nodes, distant metastases, as well as peritoneal and bone metastases in upper-abdominal tumors such as pancreatic, liver, and gastric cancers. In addition, compared with [^18^F]FDG-PET/CT, it shows a significant advantage in diagnostic sensitivity among these patients.

## Figures and Tables

**Table 1:** Characteristics of the literature included in the study.

Study Name (Author/Year)	Type	No. Patient	Country of Correspondence	Age (Years)	Ratio (M/F)	Radioactive Tracers	Imaging Method	Image Analysis	Cancer Type	Interval Time (Days)	Diagnostic Method	Imaging Values
Pang Y 2021 [21]	R	36	China	62.1 ± 10.1	25/11	[^68^Ga]Ga-DOTA-FAPI-04 vs. [^18^F]F-FDG	PET/CT	V + Sq	PC	≤7	Path + Surg + Img FU	*
Guo W 2021 [24]	R	34	China	60.6	25/9	[^68^Ga]Ga-DOTA-FAPI-04 vs. [^18^F]F-FDG	PET/CT	V + Sq	HCC + ICC	≤7	Path + Surg + Img FU	*
Qin C 2021 [29]	P	20	China	56.0	9/11	[^68^Ga]Ga-DOTA-FAPI-04 vs. [^18^F]F-FDG	PET/CT, PET/MR	V + Sq	GC	≤7	Path + Surg + Img FU	SUV_max_
Pang Y 2021 [30]	R	35	China	64.0	18/17	[^68^Ga]Ga-DOTA-FAPI-04 vs. [^18^F]F-FDG	PET/CT	V + Sq	GC	≤7	Path + Surg + Img FU	*
Wang H 2021 [31]	R	25	China	59.4 ± 6.9	24/1	[^68^Ga]Ga-DOTA-FAPI-04 vs. [^18^F]F-FDG	PET/CT	V + Sq	HCC	≤7	Path + Surg + Img FU	SUV_max_
Jiang D 2022 [28]	R	38	China	63.7 ± 15.3	29/9	[^68^Ga]Ga-DOTA-FAPI-04 vs. [^18^F]F-FDG	PET/CT	V + Sq	GC	≤2	Path + Surg + Img FU + Gastro	SUV_max_
Kuten J 2022 [32]	P	13	Israel	70.0	6/7	[^68^Ga]Ga-DOTA-FAPI-04 vs. [^18^F]F-FDG	PET/CT	V + Sq	GC	≤23	Path + Surg + Img FU	*
Lin R 2022 [33]	P	56	China	63.8 ± 14.9	40/16	[^68^Ga]Ga-DOTA-FAPI-04 vs. [^18^F]F-FDG	PET/CT	V + Sq	GC	≤7	Path + Surg + Img FU + Gastro	SUV_max_
Zhang Z 2022 [34]	P	30	China	*	17/13	[^68^Ga]Ga-DOTA-FAPI-04 vs. [^18^F]F-FDG	PET/CT, PET/MR	V + Sq	PC	≤7	Path + Surg + Img FU + Gastro	SUV_max_
Zhang S 2022 [35]	R	25	China	56.0 ± 12.0	12/13	[^68^Ga]Ga-DOTA-FAPI-04 vs. [^18^F]F-FDG	PET/CT	V + Sq	GC	≤7	Path + Surg + Img FU + Gastro	SUV_max_
Wu C 2022 [36]	R	35	China	54.0	21/14	[^18^F]ALF-NOTA-FAPI-74 vs. [^18^F]F-FDG	PET/CT	V + Sq	GC	≤7	Path + Surg + Img FU	*
Liu Q 2023 [19]	R	51	China	61.7	33/18	[^68^Ga]Ga-DOTA-FAPI-04 vs. [^18^F]F-FDG	PET/CT	V + Sq	PC	≤5	Path + Surg + Img FU	SUV_max_
Ding J 2023 [20]	P	49	China	60.9 ± 8.9	26/23	[^68^Ga]Ga-DOTA-FAPI-04 vs. [^18^F]F-FDG	PET/CT	V + Sq	PC	*	Path + Surg + Img FU	SUV_max_
Li JH 2023 [22]	P	47	China	59.1 ± 11.0	21/26	[^68^Ga]Ga-DOTA-FAPI-04 vs. [^18^F]F-FDG	PET/CT	V + Sq	ICC	*	Path + Surg + Img FU	SUV_max_
Chen H 2023 [37]	R	34	China	51.0	16/18	[^68^Ga]Ga-DOTA-FAPI-04 vs. [^18^F]F-FDG	PET/CT	V + Sq	GC	≤7	Path + Surg + Img FU	*
Li C 2023 [38]	R	51	China	55.2 ± 13.1	31/20	[^68^Ga]Ga-DOTA-FAPI-04 vs. [^18^F]F-FDG	PET/CT	V + Sq	GC	≤7	Path + Surg + Img FU	*
Miao Y 2023 [39]	P	62	China	64.0	44/18	[^68^Ga]Ga-DOTA-FAPI-04 vs. [^18^F]F-FDG	PET/CT	V + Sq	GC	≤9	Path + Surg + Img FU + Gastro	*
Rajaraman V 2023 [40]	P	41	India	*	28/13	[^68^Ga]Ga-DOTA-FAPI-04 vs. [^18^F]F-FDG	PET/CT	V + Sq	HCC + ICC	≤7	Path + Img FU	*
Lyu Z 2023 [41]	P	31	China	58.2 ± 8.5	20/11	[^18^F]ALF-NOTA-FAPI-74 vs. [^18^F]F-FDG	PET/CT	V + Sq	PC	≤7	Path + Surg + Img FU	*
Zhang J 2023 [42]	P	67	China	57.0	57/10	[^18^F]F-NOTA-FAPI-04 vs. [^18^F]F-FDG	PET/CT	V + Sq	HCC	≤7	Path + Surg + Img FU	*
Kessler L 2023 [43]	P	63	Germany	59.0	33/30	[^68^Ga]Ga-DOTA-FAPI-04 vs. [^18^F]F-FDG	PET/CT	V + Sq	PC	≤28	Path + Surg + Img FU	*
Zhang ZY 2024 [17]	P	31	China	60.2 ± 8.8	18/13	[^68^Ga]Ga-DOTA-FAPI-04 vs. [^18^F]F-FDG	PET/CT, PET/MR	V + Sq	PC	≤14	Path + Surg + Img FU	*
Li X 2024 [18]	P	62	China	63.0	43/19	[^18^F]F-NOTA-FAPI-04 vs. [^18^F]F-FDG	PET/CT	V + Sq	PC	≤14	Path + Surg + Img FU	*
Liang J 2024 [23]	P	23	China	61.0	17/6	[^18^F]F-NOTA-FAPI-04 vs. [^18^F]F-FDG	PET/CT	V + Sq	ICC	≤14	Path + Surg + Img FU	*
Liang Z 2024 [25]	P	44	China	59.3 ± 12.0	40/4	[^18^F]F-NOTA-FAPI-04 vs. [^18^F]F-FDG	PET/CT	V + Sq	HCC + ICC	≤7	Path + Surg + Img FU	SUV_max_
Lv J 2024 [26]	R	65	China	54.0 ± 10.4	26/39	[^18^F]ALF-NOTA-FAPI-04 vs. [^18^F]F-FDG	PET/CT	V + Sq	GC	*	Path + Surg + Img FU + Gastro	*
Yang J 2024 [27]	R	47	China	52.3 ± 10.2	22/25	[^18^F]ALF-NOTA-FAPI-04 vs. [^18^F]F-FDG	PET/CT	V + Sq	GC	≤14	Path + Surg + Img FU	SUV_max_
Zhang Z 2024 [44]	R	44	China	64.0	28/16	[^68^Ga]Ga-DOTA-FAPI-04 vs. [^18^F]F-FDG	PET/CT	V + Sq	ICC	≤7	Path + Surg + Img FU	*
Xu W 2024 [45]	P	112	China	*	76/36	[^18^F]ALF-NOTA-FAPI-74 vs. [^18^F]F-FDG	PET/CT	V + Sq	HCC + GC + PC	≤7	Path + Surg + Img FU	*
Yun WG 2024 [46]	P	20	Korea	69.0	8/12	[^18^F]ALF-NOTA-FAPI-74 vs. [^18^F]F-FDG	PET/CT	V + Sq	PC	*	Path + Surg + Img FU	*
Sun Y 2024 [47]	P	86	China	62.0	37/49	[^68^Ga]Ga-DOTA-FAPI-04 vs. [^18^F]F-FDG	PET/CT	V + Sq	GC	≤7	Path + Surg + Img FU	*

The study name uses the first author’s name initials and first publication year instead of the title. The age is presented in the table as median or mean ± SD. P: prospective; R: retrospective; V: visual assessment; Sq: semi-quantitative analysis; PC: pancreatic cancer; ICC: intrahepatic cholangiocarcinoma; HCC: hepatocellular carcinoma; GC: gastric cancer. The symbol ‘*’ in the table indicates that the data are missing.

## Data Availability

No new data were created or analyzed in this study. Data sharing is not applicable to this article.

## Supplementary Materials

The following supporting information can be downloaded at: https://www.mdpi.com/article/10.3390/diagnostics16040520/s1. Figure S1: Risk of bias assessment summary (QUADAS-2); Figure S2: Assessment of publication bias; Figure S3: Sensitivity analysis (based on patient data); Figure S4: Sensitivity analysis (based on lesions data); Figure S5: Sensitivity analysis (based on imaging SUV_max_ data); Table S1: Search strategy table; Table S2: Diagnostic data (based on patient counts); Table S3: Diagnostic data (based on lesions counts); Table S4: Imaging SUV_max_ data of the lesion; Table S5: PRISMA Checklist [60].

## Abbreviations

The following abbreviations are used in this manuscript:

FAPI	Fibroblast activation protein inhibitors
[^18^F]FDG	[^18^F]fluoro-2-deoxy-D-glucose
PET/CT	Positron emission tomography/computed tomography
FAP	Fibroblast activation protein
SUV_max_	Standardized uptake values maximum
QUADAS-2	Quality Assessment of Diagnostic Accuracy Studies 2
