# Supplementary material for: Diagnostic Efficacy of FAPI-PET/CT Versus [18F]FDG-PET/CT in Upper-Abdominal Malignancies: A Systematic Review and Meta-Analysis
Source: Diagnostics (Basel). 2026 Feb 9;16(4):520. doi: 10.3390/diagnostics16040520 (PMC12940046; doi:10.3390/diagnostics16040520)
Supplement: Supplementary file 1 [file diagnostics-16-00520-s001.zip › Supplementary Figure S1.pdf]

Supplementary Figure S1

Study	Risk of Bias				Applicability Concerns			Overall
	Patient Selection	Index Test	Reference Standard	Flow and Timing	Patient Selection	Index Test	Reference Standard	
Chen H 2023[37]	+	+	+	+	+	?	?	+
Ding J 2023[20]	+	+	-	?	+	+	?	-
Guo W 2021[24]	+	+	+	+	+	?	+	+
Jiang D 2022[28]	+	+	+	+	+	+	+	+
Kuten J 2022 [32]	+	+	+	?	+	?	?	-
Kuten J 2023[43]	+	+	+	-	+	?	?	-
Li C 2023[38]	+	+	+	+	+	?	+	+
Li JH 2023[22]	+	+	?	?	+	+	?	-
Li X 2024[18]	+	+	+	+	+	?	+	+
Liang J 2024[23]	+	+	+	+	+	+	+	+
Liang Z 2024[25]	+	+	+	+	+	+	+	+
Lin R 2022[33]	+	+	+	+	+	+	+	+
Liu Q 2023[19]	+	+	+	+	+	+	+	+
Lv J 2024[26]	+	+	+	-	+	?	?	-
Lyu Z 2023[41]	+	+	+	?	+	?	?	-
Miao Y 2023[39]	+	+	+	+	+	?	+	+
Pang Y 2021[21]	+	+	+	+	+	?	+	+
Pang Y 2021[30]	+	+	+	+	+	?	+	+
Qin C 2021[29]	+	+	+	+	+	+	+	+
Rajaraman V 2023[40]	-	+	+	+	?	?	+	-
Sun Y 2024[47]	+	+	+	+	+	?	+	+
Wang H 2021[31]	-	+	+	+	?	+	?	-
Wu C 2022[36]	+	-	+	+	+	?	?	-
Xu W 2024[45]	-	+	+	?	+	?	+	-
Yang J 2024[27]	+	+	+	+	+	+	+	+
Yun WG 2024[46]	+	+	+	-	+	?	?	-
Zhang J 2023[42]	+	+	+	+	+	?	+	+
Zhang S 2022[35]	+	+	+	+	+	+	+	+
Zhang Z 2022[34]	-	+	+	+	?	+	?	-
Zhang Z 2024[44]	+	+	+	+	+	?	+	+
Zhang ZY 2024[17]	+	+	?	+	+	?	?	-

Risk category :

- + low risk
- - medium risk
- * high risk
- ? information unclear

Figure S1: Assessment of the risk of bias in the literature included in the systematic review**.** The risk of bias comprises four domains—patient selection, index test, reference standard, and flow and timing—and includes three applicability concern indicators, for a total of seven data items.
