# Supplementary material for: Diagnostic Efficacy of FAPI-PET/CT Versus [18F]FDG-PET/CT in Upper-Abdominal Malignancies: A Systematic Review and Meta-Analysis
Source: Diagnostics (Basel). 2026 Feb 9;16(4):520. doi: 10.3390/diagnostics16040520 (PMC12940046; doi:10.3390/diagnostics16040520)
Supplement: Supplementary file 1 [file diagnostics-16-00520-s001.zip › Supplementary Figure S2.pdf]

Supplementary Figure S2

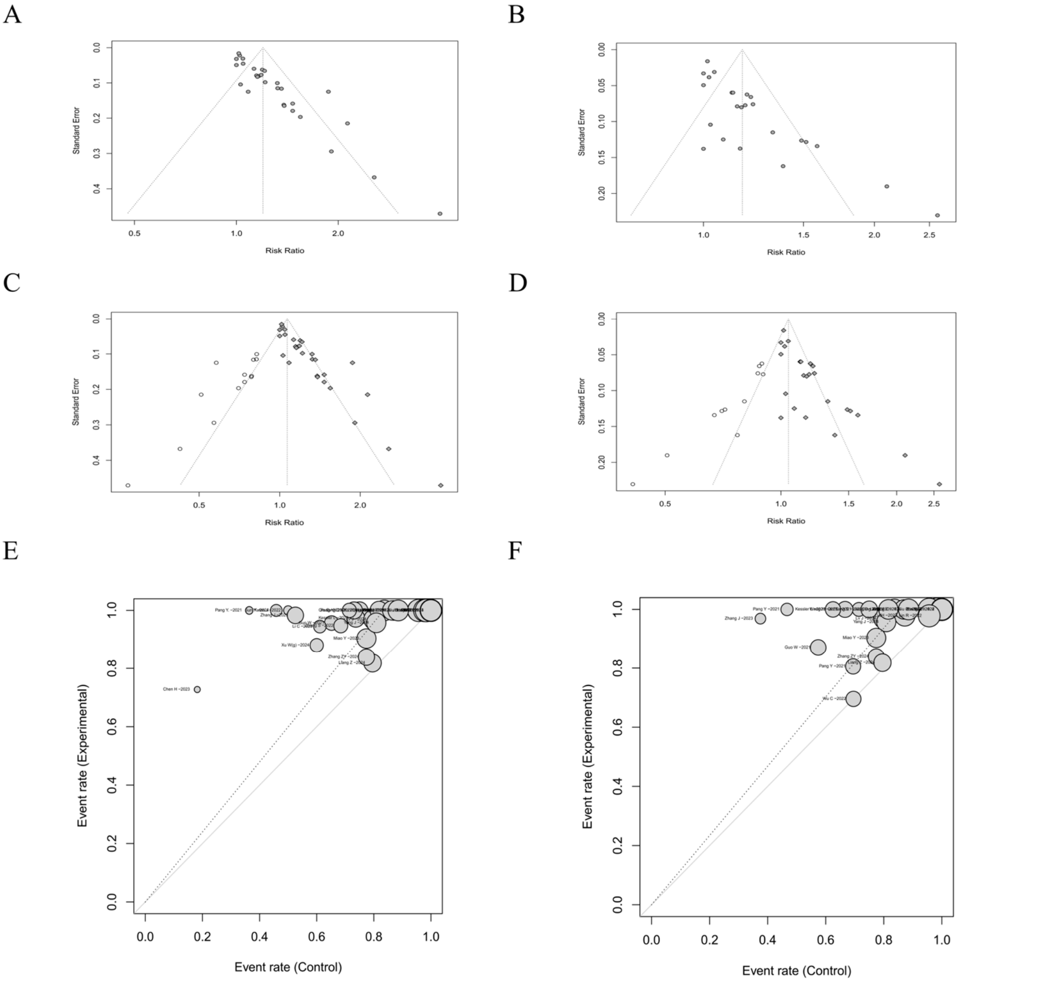

Figure S2: Assessment of the risk of bias in the sample data included in the study. Publication bias funnel plots for the number of patients (**A**) and lesions in included literature (**B**); publication bias funnel correction plots for the number of patients (**C**) and lesions (**D**); The L'Abbé plots comparing FAPI-PET/CT and [18F]FDG-PET/CT based on the number of patients (**E**) and lesions (**F**), with circle size indicating sample size.
