# Supplementary material for: Diagnostic Efficacy of FAPI-PET/CT Versus [18F]FDG-PET/CT in Upper-Abdominal Malignancies: A Systematic Review and Meta-Analysis
Source: Diagnostics (Basel). 2026 Feb 9;16(4):520. doi: 10.3390/diagnostics16040520 (PMC12940046; doi:10.3390/diagnostics16040520)
Supplement: Supplementary file 1 [file diagnostics-16-00520-s001.zip › Supplementary Figure S5.pdf]

Supplementary Figure S5

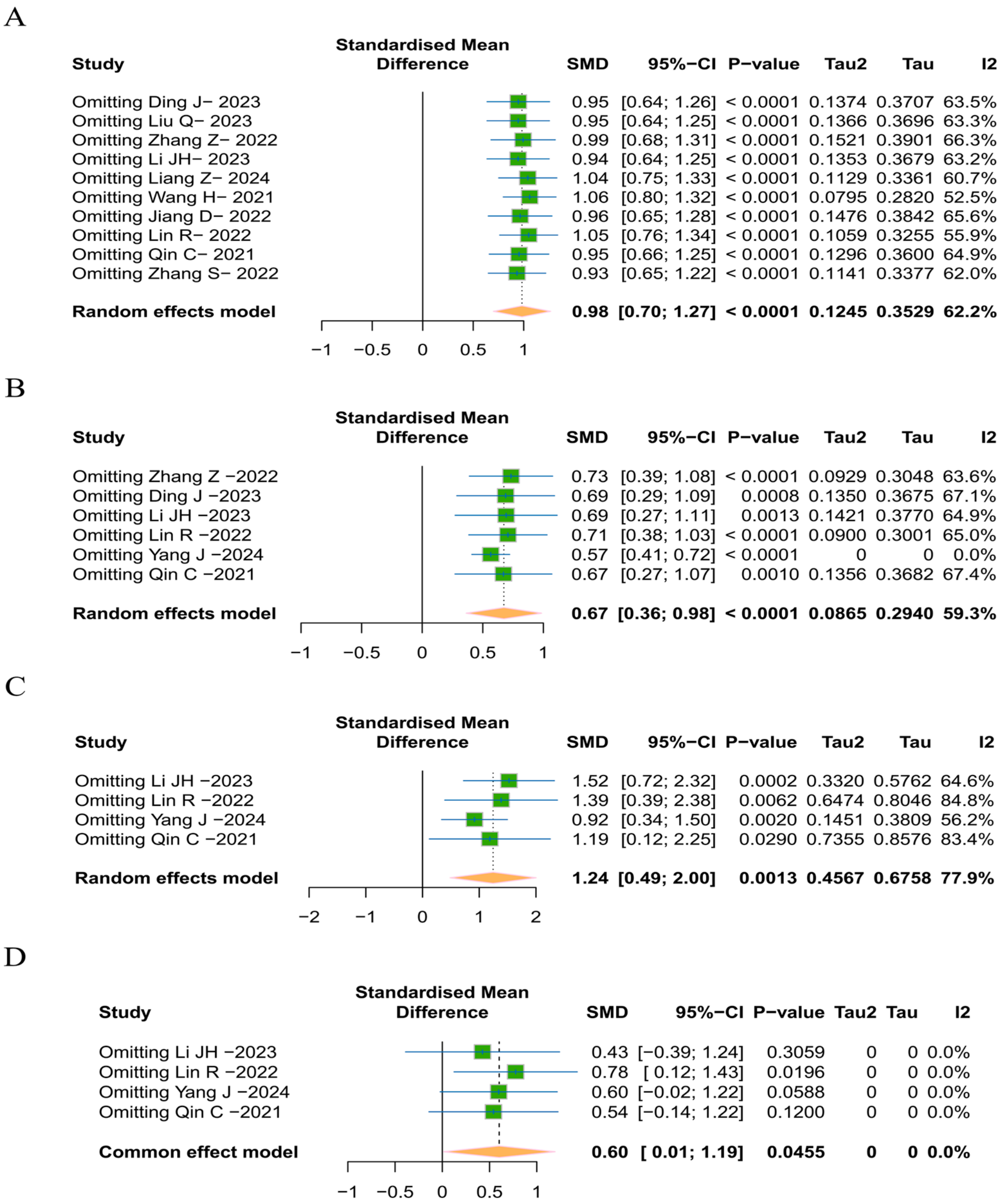

Figure S5: Sensitivity analysis based on lesion SUV_{max} data for FAPI-PET/CT and [^{18}F]FDG-PET/CT, tumor lesions (**A**), lymph node metastatic lesions (**B**), peritoneal metastatic lesions (**C**) and bone metastatic lesions (**D**)
