# Supplementary material for: Diagnostic Efficacy of FAPI-PET/CT Versus [18F]FDG-PET/CT in Upper-Abdominal Malignancies: A Systematic Review and Meta-Analysis
Source: Diagnostics (Basel). 2026 Feb 9;16(4):520. doi: 10.3390/diagnostics16040520 (PMC12940046; doi:10.3390/diagnostics16040520)
Supplement: Supplementary file 1 [file diagnostics-16-00520-s001.zip › Supplementary Table S2.pdf]

Supplementary Table S2. Diagnostic Data (Based on Patient Counts)

Diagnostic Data (Number of patients)																
**Title**		**FAPI-PET/CT**					**$[^{18}F]$FDG-PET/CT**					**Total**				
Study	Cancer type	Tumor	Lymph node	Distant metastasis	Peritoneum	Bone	Tumor	Lymph node	Distant metastasis	Peritoneum	Bone	Tumor	Lymph node	Distant metastasis	Peritoneum	Bone
**Pang Y 2021**	PC	26	23	23	10	6	19	16	18	7	3	26	23	23	10	6
**Guo W 2021**	HCC+ICC	22	16	*	3	5	15	16	*	3	5	23	16	*	3	5
**Qin C 2021**	GC	14	12	29	10	3	10	10	23	4	3	14	12	30	10	3
**Pang Y 2021**	GC	11	*	*	*	*	4	*	*	*	*	11	*	*	*	*
**Jiang D 2022**	GC	24	6	2	*	*	18	5	0	*	*	24	10	2	*	*
**Kuten J 2022**	GC	10	*	*	*	*	5	*	*	*	*	10	*	*	*	*
**Lin R 2022**	GC	45	5	*	13	4	44	5	*	9	4	45	11	41	13	4
**Zhang S 2022**	GC	18	*	*	*	*	13	*	*	*	*	19	*	*	*	*
**Liu Q 2023**	PC	46	*	25	*	*	44	*	21	*	*	46	*	25	*	*
**Ding J 2023**	PC	49	9	9	2	1	41	5	5	0	0	49	16	9	2	1
**Li JH 2023**	ICC	22	25	14	8	8	21	25	14	8	8	22	25	14	8	8
**Chen H 2023**	GC	16	*	*	*	*	4	*	*	*	*	22	*	*	*	*
**Li C 2023**	GC	17	9	22	28	3	11	6	16	17	3	18	11	23	28	3
**Miao Y 2023**	GC	56	15	*	11	2	48	13	*	5	3	62	20	*	12	3
**Lyu Z 2023**	PC	31	*	*	*	*	31	*	*	*	*	31	*	*	*	*
**Zhang J 2023**	HCC+ICC	58	*	*	*	*	31	*	*	*	*	59	*	*	*	*
**Kessler L 2023**	PC	37	*	*	*	*	28	*	*	*	*	38	*	*	*	*
**Zhang ZY 2024**	PC	26	14	31	3	*	24	10	28	3	*	31	17	31	3	*
**Li X 2024**	PC	62	44	28	12	3	61	40	18	11	1	62	44	28	12	3
**Liang J 2024**	ICC	23	23	23	23	*	20	23	23	23	*	23	23	23	23	*
**Liang Z 2024**	HCC+ICC	36	*	*	*	*	35	*	*	*	*	44	*	*	*	*
**Lv J 2024**	GC	63	63	61	*	*	52	50	46	*	*	65	65	65	*	*
**Yang J 2024**	GC	*	47	46	25	3	*	38	31	13	2	*	47	47	25	3
**Zhang Z 2024**	HCC+ICC	35	*	*	*	*	31	*	*	*	*	35	*	*	*	*
**Xu W 2024^{GC}**	GC	22	*	*	*	*	15	*	*	*	*	25	*	*	*	*
**Xu W 2024^{HCC}**	HCC+ICC	22	*	*	*	*	18	*	*	*	*	22	*	*	*	*
**Xu W 2024^{PC}**	PC	22	*	*	*	*	19	*	*	*	*	22	*	*	*	*
**Yun WG 2024**	PC	20	*	*	*	*	20	*	*	*	*	20	*	*	*	*
**Sun Y 2024**	GC	24	*	*	*	*	11	*	*	*	*	24	*	*	*	*

FAPI: fibroblast activation protein inhibitors; FDG: fluoro-2-deoxy-D-glucose; PC: Pancreatic cancer; ICC: Intrahepatic cholangiocarcinoma; HCC: Hepatocellular carcinoma; GC: Gastric cancer.
Xu W 2024^{GC}, Xu W 2024^{HCC} and Xu W 2024^{PC} all come from different types of cancer within the same article (Xu W 2024)
