# Supplementary material for: Diagnostic Efficacy of FAPI-PET/CT Versus [18F]FDG-PET/CT in Upper-Abdominal Malignancies: A Systematic Review and Meta-Analysis
Source: Diagnostics (Basel). 2026 Feb 9;16(4):520. doi: 10.3390/diagnostics16040520 (PMC12940046; doi:10.3390/diagnostics16040520)
Supplement: Supplementary file 1 [file diagnostics-16-00520-s001.zip › Supplementary Table S3.pdf]

Supplementary Table S3 Diagnostic Data (Based on Lesions Counts)

Diagnostic Data (Number of lesions)																
**Title**		**FAPI-PET/CT**					**[^{18}F]FDG-PET/CT**					**Total**				
Study	Cancer type	Tumor lesions	Lymph node	Distant metastasis	Peritoneum	Bone	Tumor lesions	Lymph node	Distant metastasis	Peritoneum	Bone	Tumor lesions	Lymph node	Distant metastasis	Peritoneum	Bone
**Pang Y 2021**	PC	29	144	144	77	14	25	132	79	33	7	36	169	164	77	14
**Guo W 2021**	HCC+ICC	47	32	64	12	43	31	21	55	4	33	54	32	64	12	43
**Qin C 2021**	GC	14	45	97	42	12	10	33	60	14	4	14	45	97	42	12
**Pang Y 2021**	GC	30	81	*	93	67	14	77	*	51	55	30	81	*	93	67
**Wang H 2021**	HCC+ICC	30	*	*	*	*	20	*	*	*	*	30	*	*	*	*
**Jiang D 2022**	GC	38	19	*	*	*	31	18	*	*	*	38	24	*	*	*
**Kuten J 2022**	GC	*	16	*	*	*	*	16	*	*	*	*	16	*	*	*
**Lin R 2022**	GC	45	*	*	159	64	44	*	*	47	55	46	*	*	159	64
**Zhang Z 2022**	PC	30	43	*	*	*	30	30	*	*	*	30	43	*	*	*
**Zhang S 2022**	GC	24	75	275	*	*	18	32	122	*	*	24	75	283	*	*
**Wu C 2022**	GC	32	*	85	*	*	32	*	57	*	*	46	*	106	*	*
**Liu Q 2023**	PC	46	*	139	*	*	44	*	93	*	*	46	*	139	*	*
**Ding J 2023**	PC	49	71	46	*	*	41	42	22	*	*	49	115	46	*	*
**Li JH 2023**	ICC	46	212	49	35	12	41	208	41	28	11	47	244	49	35	12
**Chen H 2023**	GC	*	464	*	59	*	*	424	*	20	*	*	493	*	59	*
**Miao Y 2023**	GC	56	*	*	*	*	48	*	*	*	*	62	*	*	*	*
**Lyu Z 2023**	PC	*	80	*	*	*	*	72	*	*	*	*	93	*	*	*
**Zhang J 2023**	HCC+ICC	207	126	168	17	*	94	115	161	14	*	226	129	195	17	*
**Kessler L 2023**	PC	32	*	*	12	6	20	*	*	4	3	32	*	*	12	6
**Zhang Z 2024**	PC	26	*	*	*	*	24	*	*	*	*	31	*	*	*	*
**Li X 2024**	PC	62	203	227	158	6	61	151	144	103	4	62	290	227	158	6
**Liang J 2024**	ICC	23	102	123	*	50	20	74	111	*	40	23	121	134	*	57
**Liang Z 2024**	HCC+ICC	36	*	*	*	*	35	*	*	*	*	44	*	*	*	*
**Lv J 2024**	GC	63	*	63	28	19	52	*	30	14	4	65	*	63	28	19
**Yang J 2024**	GC	45	47	46	25	3	38	38	31	13	2	47	47	47	25	3
**Zhang Z 2024**	HCC+ICC	35	65	107	90	*	31	52	63	26	*	35	65	172	90	*
**Xu W 2024^{GC}**	GC	*	*	*	194	*	*	*	*	58	*	*	*	*	194	*
**Xu W 2024^{HCC}**	HCC+ICC	*	*	*	8	*	*	*	*	2	*	*	*	*	8	*
**Xu W 2024^{PC}**	PC	*	*	*	41	*	*	*	*	18	*	*	*	*	41	*
**Yun WG 2024**	PC	20	*	*	*	*	20	*	*	*	*	20	*	*	*	*
**Sun Y 2024**	GC	*	156	*	*	*	*	124	*	*	*	*	173	*	*	*

FAPI: fibroblast activation protein inhibitors; FDG: fluoro-2-deoxy-D-glucose; PC: Pancreatic cancer; ICC: Intrahepatic cholangiocarcinoma; HCC: Hepatocellular carcinoma; GC: Gastric cancer.

Xu W 2024^{GC}, Xu W 2024^{HCC} and Xu W 2024^{PC} all come from different types of cancer within the same article (Xu W 2024)
