# Supplementary material for: Diagnostic Efficacy of FAPI-PET/CT Versus [18F]FDG-PET/CT in Upper-Abdominal Malignancies: A Systematic Review and Meta-Analysis
Source: Diagnostics (Basel). 2026 Feb 9;16(4):520. doi: 10.3390/diagnostics16040520 (PMC12940046; doi:10.3390/diagnostics16040520)
Supplement: Supplementary file 1 [file diagnostics-16-00520-s001.zip › Supplementary Table S4.pdf]

Supplementary Table S4: Imaging SUVmax Data of the Lesion

Lesion SUVmax Data																	
Title		Tumor lesions				Lymph node lesions				Peritoneum metastasis lesions				Bone metastasis lesions			
Study	Cancer Type	FAPI Total	FAPI Mean/sd	FDG Total	FDG Mean/sd	FAPI Total	FAPI Mean/sd	FDG Total	FDG Mean/sd	FAPI Total	FAPI Mean/sd	FDG Total	FDG Mean/sd	FAPI Total	FAPI Mean/sd	FDG Total	FDG Mean/sd
**Qin C 2021**	GC	14	11.31±3.96	10	6.18±2.46	45	8.65±4.38	33	6.13±2.79	42	8.74±3.82	14	3.71±1.53	12	10.05±6.18	4	5.20±4.56
**Wang H 2021**	HCC+ ICC	30	6.96±5.01	20	5.89±3.38	*	*	*	*	*	*	*	*	*	*	*	*
**Jiang D 2022**	GC	38	10.80±7.30	31	4.10±2.80	*	*	*	*	*	*	*	*	*	*	*	*
**Lin R 2022**	GC	45	10.3±3.80	44	8.10±4.90	5	6.30± 2.10	5	6.10±3.70	13	7.10±3.70	9	4.50±1.80	4	6.40±2.40	4	7.10±3.80
**Zhang Z 2022**	PC	30	12.58±4.44	30	8.78±3.80	23	9.35±5.42	23	7.77±4.18	*	*	*	*	*	*	*	*
**Zhang S 2022**	GC	18	10.28±4.98	13	3.20±2.51	*	*	*	*	*	*	*	*	*	*	*	*
**Liu Q 2023**	PC	46	13.50±5.80	44	7.30±3.50	*	*	*	*	*	*	*	*	*	*	*	*
**Ding J 2023**	PC	49	15.40±8.40	41	6.70±4.10	71	4.60±1.80	42	3.70±0.90	*	*	*	*	*	*	*	*
**Li JH 2023**	ICC	46	18.95±7.47	41	11.86±0.70	212	6.91±6.56	208	3.94±2.83	35	6.37±4.21	28	4.50±1.96	12	12.15±6.43	11	7.51±4.54
**Liang Z 2024**	HCC+ ICC	22	10.54±6.72	21	7.68±6.79	*	*	*	*	*	*	*	*	*	*	*	*
**Yang J 2024**	GC	*	*	*	*	47	11.45±7.59	38	3.05±2.56	25	11.93±4.80	13	2.50±1.43	3	12.40±8.45	2	5.90±3.46

FAPI: fibroblast activation protein inhibitors; FDG: fluoro-2-deoxy-D-glucose; PC: Pancreatic cancer; ICC: Intrahepatic cholangiocarcinoma; HCC: Hepatocellular carcinoma; GC: Gastric cancer
