# Supplementary material for: Diagnostic Efficacy of FAPI-PET/CT Versus [18F]FDG-PET/CT in Upper-Abdominal Malignancies: A Systematic Review and Meta-Analysis
Source: Diagnostics (Basel). 2026 Feb 9;16(4):520. doi: 10.3390/diagnostics16040520 (PMC12940046; doi:10.3390/diagnostics16040520)
Supplement: Supplementary file 1 [file diagnostics-16-00520-s001.zip › Supplementary Table S5.pdf]

Section and Topic	Item #	Checklist item	Location where item is reported
**TITLE**			
Title	1	The title has Identified the descriptions of both the systemic review and the meta-analysis.	Page 1
**ABSTRACT**			
Abstract	2	Provides a complete abstract, including: background, methods (data sources, assessment tools and registration number), results (data comparison); conclusions.	Page 1
**INTRODUCTION**			
Rationale	3	Describe the rationale for the review in the context of existing knowledge.	Page 1, Lines 73-78
Objectives	4	Provide a detailed statement of the specific objective and the comparative subjects.	Page 1, Lines 78-82
**METHODS**			
Eligibility criteria	5	All inclusion and exclusion criteria were clearly specified, and primary tumors, metastatic lymph nodes, and metastatic lesions were clearly grouped.	Page 3 Lines 93-104
Information sources	6	Specify all involved databases and search keywords. Provides a detailed explanation of the time period covered by the included literature.	Page 2,3 Lines 84-92
Search strategy	7	Present the full search strategies for all databases and websites, including any filters and limits used.	Page 2,3 Lines 84-92
Selection process	8	The specific criteria for determining whether a study meets the inclusion criteria for this review are detailed, and each reviewer conducted their work independently.	Page 3, Lines 93-104
Data collection process	9	Provides a complete explaination of three reviewers independently read the literature and completed the data collection process.	Page 3,Lines 105-111
Data items	10a	Provides a detailed description of the collected data objects.	Page 3, Lines 105-111. Supplementary table 1
	10b	Any missing data has been enumerated and presented and these data were not included in this analysis.	Page 3,4 Lines 105-136 Supplementary table 1-4

Study risk of bias assessment	11	Provides detailed information on the methods and tools used to assess the risk of bias in the included studies, as well as the working procedures of the reviewers.	Page 3,4<br>Lines 128-136
Effect measures	12	Provides a detailed description of the presentation effect measure for each result.	Page 3,4<br>Lines 128-136
Synthesis methods	13a	A detailed account of the methodological approaches applied to each data item is presented.	Page 2,3,4<br>Lines 83-136<br>Table 1
	13b	The missing data has been presented and detailed, and is excluded from the scope of this statistical analysis.	Page 3,4<br>Lines 93-136
	13c	Present the methods of each study and the comprehensive analysis in forest plot.	Page 3,4<br>Lines 112-136
	13d	A combination of random-effects and fixed-effects approaches was used for the analysis. The Cochran-Q test was employed to evaluate heterogeneity in the data. All data analyses and visualizations were conducted in R, using the meta and meta4diag packages	Page 3 ,4<br>Lines 112-136
	13e	To conduct subgroup analyses and sensitivity analyses to evaluate data.	Page 3,4<br>Lines 112-136
	13f	A leave-one-out sensitivity analysis was performed within each subgroup to evaluate the robustness of the pooled effect across subgroups.	Page 3<br>Lines 121-123
Reporting bias assessment	14	Bias risk was evaluated with the QUADAS-2 tool. Studies with missing data will undergo risk-of-bias assessment, and the missing data will not be included in this analysis.	Page 3,4<br>Lines 128-136
Certainty assessment	15	Describe any methods used to assess certainty (or confidence) in the body of evidence for an outcome.	NA

Section and Topic	Item #	Checklist item	Location where item is reported
**RESULTS**			
Study selection	16a	A flow diagram has been used to present the screening process in the manuscript.	Page 4<br>Figure 1
	16b	The screening process was described using a flow diagram to illustrate the stages and the numbers of studies included and excluded.	Page 4<br>lines 137–164<br>Figure 1
Study characteristics	17	Primary use of a tabular format to describe	Page 5,6,7,8<br>lines 137–164<br>Table 1
Risk of bias in studies	18	The original text explicitly states this and supplements it in the form of an image.	Page 4,5<br>lines 146–164<br>Supplementary Figure 1
Results of individual studies	19	For all outcome indicators, detailed effect estimates and their precision values(e.g., confidence intervals) are provided in visual formats (forest plots).	Page 9-14<br>lines 169–260<br>Figures 2–5
Results of syntheses	20a	For each synthesis, briefly summarise the characteristics and risk of bias among contributing studies.	Page 9-14<br>lines 169–260<br>Figures 2–5
	20b	All statistical syntheses should be presented in the form of forest plots. For each analysis, provide the summary estimate with its precision (e.g., confidence or credible interval) and the measures of statistical heterogeneity.	Page 9-14<br>lines 169–260<br>Figures 2–5
	20c	Present results of all investigations of possible causes of heterogeneity among study results.	NA
	20d	All sensitivity analyses conducted to assess the robustness of the synthesized results have been presented..	Supplementary Figures 2-5,<br>Supplementary tables 2–5

Reporting biases	21	Missing data were not included in the study, so no reporting bias from missing data is assessed	NA
Certainty of evidence	22	Present assessments of certainty (or confidence) in the body of evidence for each outcome assessed.	NA
**DISCUSSION**			
Discussion	23a	Provide a general interpretation of the results in the context of other evidence.	Page 14,15 lines 261–290
	23b	Discuss limitations of the evidence included in the review.	Page 15,16 lines 304–333
	23c	Discuss limitations of the review processes used.	Page 15,16 lines 304–333
	23d	Discuss implications of the results for practice and future research.	Page 15 lines 274–303
**OTHER INFORMATION**			
Registration and protocol	24a	This registration protocol is initiated via an online application and gets approval.	Page 2 lines 85–86
	24b	The protocol was not prepared.	NA
	24c	No modifications were made to the study registration.	NA
Support	25	This research received no external funding	Page 16 Line 343
Competing interests	26	The manuscript reports no conflicts of interest.	Page 16 lines 350–351
Availability of data, code and other materials	27	All relevant data and information are provided within the manuscript and supplementary mate-rials, including references to the original studies.	Page 16 lines 346–347

From: Page MJ, McKenzie JE, Bossuyt PM, Boutron I, Hoffmann TC, Mulrow CD, et al. The PRISMA 2020 statement: an updated guideline for reporting systematic reviews. BMJ 2021;372:n71. doi: 10.1136/bmj.n71. This work is licensed under CC BY 4.0. To view a copy of this license, visit https://creativecommons.org/licenses/by/4.0/
